# Left Ventricular Response to Cardiac Resynchronization Therapy: Insights From Hemodynamic Forces Computed by Speckle Tracking

**DOI:** 10.3389/fcvm.2019.00059

**Published:** 2019-05-14

**Authors:** Matteo Dal Ferro, Valerio De Paris, Dario Collia, Davide Stolfo, Thomas Caiffa, Giulia Barbati, Renata Korcova, Bruno Pinamonti, Luigino Zovatto, Massimo Zecchin, Gianfranco Sinagra, Gianni Pedrizzetti

**Affiliations:** ^1^Cardiovascular Department, Azienda Ospedaliera Universitaria Integrata of Trieste, Trieste, Italy; ^2^Department of Engineering and Architecture, University of Trieste, Trieste, Italy; ^3^Biostatistics Unit, Department of Medical Sciences, University of Trieste, Trieste, Italy

**Keywords:** cardiac resynchronization therapy, deformation imaging, hemodynamic forces, intraventricular pressure gradient, cardiac mechanics, speckle tracking echocardiography

## Abstract

**Aims:** Despite continuous efforts in improving the selection process, the rate of non-responders to cardiac resynchronization therapy (CRT) remains high. Recent studies on intraventricular blood flow suggested that the alignment of hemodynamic forces (HDFs) may be a reproducible biomarker of mechanical dyssynchrony. We aimed to explore the relationship between pacing-induced realignment of HDFs and positive response to CRT.

**Methods and results:** We retrospectively analyzed 38 patients from the CRT database of our institution fulfilling the inclusion criteria for HDFs-related echocardiographic assessment early pre and post CRT implantation, with available mid-term follow-up (≥ 6 months) evaluation. Standard echocardiographic and deformation parameters early pre and post CRT implantation were integrated with the measurement of HFDs through novel methods based on speckle-tracking analysis. At midterm follow-up 71% of patients were classified as responders (reduction of Left Ventricular Systolic Volume Indexed ≥ 15%). Patients did not display significant changes between close evaluations pre and post-implant in terms of ejection fraction and strain metrics. A significant reduction of the ratio between the amplitudes of transversal and longitudinal force components was found. The variation of this ratio strongly correlates (R^2^ =0.60) with Left Ventricular (LV) end-systolic volume variation at mid-term follow up.

**Conclusion:** Pacing-induced realignment of HDFs is associated with CRT efficacy at follow up. These preliminary results claim for dedicated prospective clinical studies testing the potential impact of HDFs study for patient selection and pacing optimization in CRT.

## Introduction

Cardiac resynchronization therapy (CRT) is an established strategy for patients with symptomatic heart failure (HF), reduced systolic function and prolonged QRS duration, improving symptoms and long-term survival (1–4). Nevertheless, the effect of CRT is not homogeneous ranging between the complete recovery of LV function to absent or only trivial changes in LV geometry and contractility (5). Despite strong efforts in the identification of the appropriate candidates to CRT, the understanding of the pathophysiological basis underlining the response to CRT is largely incomplete and the rate of non-responders remains high (5). This observation emphasizes the need for further studies to improve patient selection.

Electrocardiographic criteria are considered the most reliable for eligibility to CRT. However, a heterogeneity of CRT response is found even in carefully selected patients with LBBB morphology and QRS duration >120 ms. Therefore, imaging markers of mechanical dyssynchrony were considered as adjunctive criteria for patient selection; however, results were still heterogeneous in wide QRS patients, and even negative in narrow QRS ones (6).

Hemodynamic Forces (HDFs), or equivalently intraventricular pressure gradients (IVPGs), are a relatively novel imaging marker of LV function. HDFs drive blood motion during both ventricular ejection and ventricular filling, and are deeply interconnected with physiological vortex formation inside the LV (7). Under normal conditions, LV HDFs present a dominant longitudinal (base-apex) orientation (8–10). Recent studies demonstrated that a reduction of the longitudinal component with a relative increase of the transversal ones represent a sensible marker of dyssynchrony (11–13) and a preliminary echocardiographic study suggested that that the CRT-induced restoration of the normal orientation of LV HDFs is associated with LV reverse remodeling (14).

Therefore, in the present study we sought (A) to describe the pattern of HDFs in reduced ejection fraction HF (HFrEF) patients with wide QRS, (B) to assess early CRT-induced changes in HDFs and (C) to assess the potential relationship between CRT-induced changes in HDFs orientation and mid-term clinical response.

## Methods

### Patient Selection and Study Design

We retrospectively analyzed all patients with successful CRT device implantation consecutively included in the CRT-Registry of our Institution (an ongoing research database prospectively including all patients undergoing CRT) from January 2008 to December 2016. Follow-up ended on 31 June 2017. Indication for CRT was symptomatic HF with LV ejection fraction (LVEF) ≤35% and QRS duration ≥120 ms, despite optimal medical therapy. The study received institutional review board approval, and informed consent was obtained under the institutional review board policies of the hospital administration. Ischemic etiology of HF was considered in the presence of significant CAD (stenosis >50% of a major coronary artery on coronary angiography) and/or history of prior myocardial infarction or revascularization.

According to institutional protocol the majority of patients implanted underwent complete echocardiographic examination prior and after CRT implantation. Inclusion criteria for the present study were: (1) available complete transthoracic echocardiographic examinations within a close time range of 2 months before and after CRT implantation; (2) available complete transthoracic echocardiographic examination at mid-term follow-up (~6 months). Patients with poor quality imaging, in particular not allowing the complete tracking of endocardial profile in 4-, 2-, and 3-chamber apical view along with optimal LVOT and aortic valve visualization, or with insufficient frame rate (<50 Hz) for speckle tracking analysis, were excluded.

All the metrics of interest, in particular HDFs, were analyzed retrospectively in the last echo within 2 months before CRT implantation (PRE) and in the first echo within 2 months after implant (POST). CRT-induced changes in main parameters [LVEF, LV End Systolic and End Diastolic Volume Indexed (LVESVI and LVEDVI), end-systolic Global Longitudinal Strain (GLS), Standard Deviation of Time To Peak of Longitudinal Strain (SD_TTP_LS)] and in HDFs, where compared between PRE and POST, and subsequently correlated with LV volumetric remodeling at follow up. LV remodeling at follow up was defined, as previously described (15), by relative changes in LV End Systolic Volume (LVESV) [(follow-up LVESV—PRE LVESV)/PRE LVESV^*^100)] and categorized as follows: super-responders (SR) patients had a LVESV reduction ≥30%; responders (R) had a LVESV reduction ranging between 29 and 15%, non-responders (NR) had a LVESV reduction ranging between 14 and 0% and finally negative-responders (NegR) had an increase in LVESV.

The HDFs-related metric which resulted significantly correlated with LV remodeling at midterm follow up, was finally selected for prediction test of patient's responder status (intended equivalently as a LV volume reduction ≥15% and/or R/SR category) (see *Statistical analysis for further details)*.

### Device Implantation

CRT devices from major manufacturers (Biotronik, Guidant-Boston Scientific, Medtronic, Livanova-Sorin, and St. Jude Medical) were used. Unipolar or bipolar leads were implanted in posterolateral or lateral veins when feasible. Ablation of the atrioventricular node after the procedure was considered for patients with an unsatisfactory proportion of ventricular pacing (<95%).

### Echocardiographic Analyses

Echocardiograms were recorded on digital media storage devices at the echocardiographic core laboratory of our Institution and analyzed offline according to current international guidelines (16).

The three apical echocardiographic recordings (4-, 2-, and 3-chamber) were re-analyzed offline by a prototype version, that includes the evaluation of HDFs, of a commercially available speckle tracking solution (2DCPA v.1.4; TomTec Imaging Systems Gmbh, Unterschleissheim, Germany). The software requires drawing the end-systolic (ES) endocardial borders and tracks the endocardial border over the entire heartbeat; it then allows correcting the end-diastolic (ED) border and propagates the correction over the entire cycle to match the original ES border. The ES and ED volumes are computed by the Simpson method, the GLS is computed as by guidelines (17); then a measure of mechanical synchrony is evaluated by the standard deviation among the 16 segments of the time to peak of longitudinal strain segmental curves (SD_TTP_LS).

From the three endocardial borders estimated by speckle tracking during the cardiac cycle the software also computes the HDFs. The calculation procedure is described in detail in Pedrizzetti et al. (18), which reports also the validation with respect to 4D Flow MRI measurements. Briefly, the total HDF vector, ***F***(*t*), exchanged between blood and tissues at every instant during the cardiac cycle is evaluated by the balance of momentum inside the LV volume *V*(*t*)

$$\begin{array}{l} F\left(t\right)=\int_{S\left(t\right)}\rho vv_{n}dS+\int_{V\left(t\right)}\rho \frac{\partial v}{\partial t}dV, \end{array}$$

where *S*(*t*) is the surface bounding the volume, ***v*** is the velocity vector and the subscript *n* indicates the outward normal component. The first term in the right-hand side of the previous formula represents the flux of momentum across the instantaneous LV volume boundary. This is computed from the velocity at the LV endocardium, given by speckle tracking, and by the mean velocity across the mitral and aortic valves that is evaluated by the LV volume rate divided by the valve area. The second term, due to blood inertia, is obtained by the rate of change of the average velocity inside the LV, which can be reliably estimated through simple assumptions on the global intraventricular fluid dynamic (18). The time profile of the longitudinal component (*F*_*z*_(*t*), apex-to-base) and of the main transversal component (*F*_*x*_(*t*), inferolateral-anteroseptal) are thus obtained, and they are normalized with the instantaneous LV volume, and the blood specific weight, to have a dimensionless number not directly affected by the chamber size.

The global amplitude of the individual HDF components is described in terms of the root mean square (RMS) value of the corresponding time profile during the heartbeat period *T*

$$\begin{array}{l} \begin{array}{cc} RMSz=\sqrt{\frac{1}{T}\int_{0}^{T}F_{z}^{2}\left(t\right)dt}, & RMSx=\sqrt{\frac{1}{T}\int_{0}^{T}F_{x}^{2}\left(t\right)dt}. \end{array} \end{array}$$

The entity of the deviation of the HDF from the longitudinal direction is estimated by the ratio of the amplitudes: RMSx/RMSz.

### Statistical Analysis

Summary statistics of clinical and instrumental variables were expressed as the mean and standard deviation (SD), median and interquartile range (IQR), or counts and percentage, as appropriate.

The longitudinal evolution of the parameters under study from PRE- to POST- CRT examination was assessed by the two-tailed paired Student *t*-test or Wilcoxon test according to Gaussian or Non Gaussian distribution, with significance level taken as *p* < 0.05.

Mechanical echocardiographic variables, and their changes from PRE- to POST-CRT were correlated (Pearson test) with the relative changes in LVESV (ΔLVESV), where Δ indicated the difference from PRE to mid-term follow-up, divided by PRE LVESV.

Correlations were graded by the coefficient of determination R^2^, with significance threshold, *R*^2^ > 0.4. A receiver operating characteristic (ROC) curve analysis was performed to determine the best cutoff value (according to Youden criterion) of RMSx/RMSz PRE, RMSx/RMSz POST, and δ(RMSx/RMSz), where δ indicated the change from PRE to POST, for differentiating “all grade” responders (including both R and SR, i.e., with LVESV reduction at midterm follow up ≥ 15%) and “all grade” non-responders (NR and NegR, LVESV reduction at midterm follow up < 15%).

The Prism Software (GraphPad) version 5 was used for descriptive analyses, and the Statistical processing was performed with MatLab (Natick, MA, USA; MatLab R2014a, ver. 8.3.0.532 with Statistics Toolbox R2014aR).

### Reproducibility of HDFs Analysis

Inter-operator variability of HDFs quantification was evaluated by repeating evaluation on the same images by a second operator, blind to the previous results, on all 38 patients both on echo PRE and POST CRT (76 triplane samples). This allowed to obtain a power of 80% to identify an intraclass correlation coefficient (ICC) of 0.70, under the null hypothesis of ICC equal to 0.50, using an F test at a level of significance equal to 5% and intra-operator ICC of 0.80 under the null hypothesis of 0.60. The differences were described in terms of average with 95 confidence interval; statistical comparison was also verified by a paired two-tailed Student *t*-test with significance level assumed *p* = 0.05. Bland—Altman analysis was also performed to verify the absence of systematic bias.

## Results

### Study Population Characteristics

Of the about 300 patients included in CRT registry in the selected period, only 38 fulfilled inclusion criteria. Table 1 shows main clinical and echocardiographic characteristics of the study population at PRE-CRT (median time 12 days before implantation, IQR 6-46) and at midterm follow up (median time 7.3 months after implantation, IQR 4-10.5). Basal QRS duration was 166 ± 24 ms, and LBBB morphology was present in 97% of patients. Comprehensively, both LVEDV and LVESV decreased at mid-term follow-up with a coherent improvement in LVEF, with 71% of our patients classified as R or SR, whereas the remaining 29% were classified NR or NegR, according to the abovementioned classification. QRS duration, in parallel, decreased at 156 ± 23 ms.

**Table 1:** Population characteristics and main echocardiographic data at basal and midterm follow up.

**Population baseline characteristics**	**Total (*n* = 38)**
Age	64 (37–83)
Male(%)	28 (73%)
Ischemic etiology(%)	10 (26%)
LBBB (%)	37 (97%)
QRS duration (ms)	166 ± 24
AF (%)	9 (23%)
NYHA class III-IV(%)	16 (42%)
SBP (mmHg)	119 ±18
ACE inhibitors/ARBs therapy (%)	34 (89%)
Beta blocker therapy (%)	32 (84%)
MRA therapy (%)	20 (52%)
Loop diuretics (%)	37 (97%)
HF duration (months)	76 (35–136)
**ECHOCARDIOGRAPHIC BASELINE (PRE) CHARACTERISTICS**	
LVEDVI (ml/m2)	118 (97–164)
LVESVI (ml/m2)	92 (73–127)
LVEF (%)	22 ± 6
MR ≥ 2+ (%)	27 (71%)
**POPULATION CHARACTERISTICS AT MIDTERM FOLLOW UP**	
Time to Echo at follow up (months)	7.3 (4–10.5)
LVEDVI (ml/m2)	96 (76–124)^*^
LVESVI (ml/m2)	67 (48–95) #
LVEF (%)	33 ± 11§
NYHA class III-IV(%)	8 (21%)
MR ≥ 2+ (%)	21 (55%)
QRS duration (ms)	156 ± 23
Ventricular Pacing (%)	97 ± 3
SR/R/NR/NegR	17 (45%) /10 (26%) /5 (13%) /6 (16%)

*Values are expressed as the mean ± SD or median with interquartile range, and as a percentage (%)*.

LVEDVI, left ventricular end-diastolic volume indexed; LVESVI, left ventricular end-systolic volume indexed; LVEF, left ventricular ejection fraction; MR, mitral regurgitation; SBP, systolic blood pressure; GLS, global longitudinal strain;

* *p < 0,0001 with LVEDVI basal; #p < 0,0001 with LVESVI basal, §p < 0,0001 with LVEF basal, SR, Super responders; R, Responders; NR, Non responders; NegR, Negative responders*.

**Figure 1:**
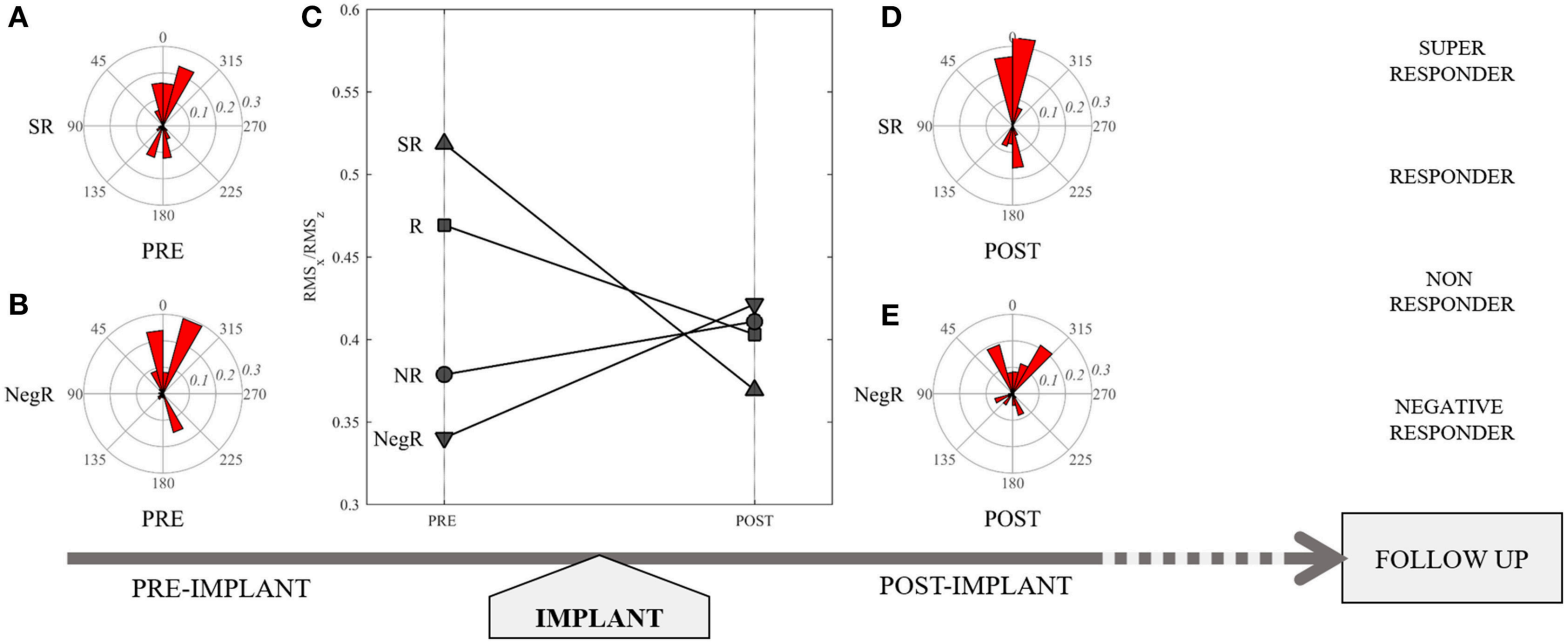
Representation of study design: **(C)** patient's categorization according to different values of RMSx/RMSz PRE CRT implant, and their variation POST CRT, which resulted representative of responder status at follow up. Polar representation of the distribution of hemodynamic forces in a Super Responder (SR) patient pre **(A)** and post **(D)**, and in a Negative Responder (NegR) pre **(B)** and post **(E)** CRT implant.

In Table 2 the main echocardiographic parameters assessed at PRE-CRT examination were compared with POST-CRT examination (median time 3 days post-implantation, IQR 2-9). The LV volumes present a quantitatively small but statistically significant (*p* < 0.001 for both) reduction from PRE to POST. The other variables, LVEF, GLS, SD_TTP_LS and the amplitude (RMS) of HDF force components did not present significant changes; however, a small increase in GLS and in the value of RMSz was noticed POST implant. Most importantly, the ratio between transversal and longitudinal HDFs (RMSx/RMSz) shows a significant reduction POST CRT (*p* = 0.011). This ratio was selected for further evaluation: with respect to the evaluation of single RMSz or RMSx taken separately, their combined evaluation expressed by this ratio seems to offer a more accurate representation of the degree of alignment of LV IVPGs distribution in disease state. Figure 1 highlights the role of this parameter in the study design.

**Table 2:** Comparison of early changes in main echocardiographic parameters after CRT implant.

	**Last echo PRE CRT**	**Early first echo POST CRT**	***p*-value**
Time from last echo to CRT (days)	12 (6–46)	—	—
Time from CRT to echo (days)	—	3 (2–9)	—
LVEDVI (ml/m2)	118 (97–164)	112 (83–143)	*p* = 0.0006
LVESVI (ml/m2)	92 (73–127)	80 (65–115)	*p* = 0.0005
LVEF (%)	22 ± 6	22 ± 8	NS
GLS	−5.8 ± 2.5	−6.4 ± 3.0	NS
SD_TTP_LS	14.2 ± 5.9	14.0 ± 6.5	NS
RMSz (Apex-Base)	0.058 ± 0.040	0.064 ± 0.033	NS
RMSx (iLat-aSept)	0.024 ± 0.013	0.023 ± 0.010	NS
RMSx/RMSz	0.46 ± 0.18	0.39 ± 0.12	*p* = 0.0107

*Values are expressed as the mean ± SD or median with interquartile (IQR) range*.

*LVEDVI, left ventricular end-diastolic volume indexed; LVESVI, left ventricular end-systolic volume indexed; LVEF, left ventricular ejection fraction; GLS, global longitudinal strain; SDTTPLS, standard deviation time to peak of longitudinal strain; RMSz, root mean square of longitudinal hemodynamic force; RMSx, root mean square of transversal hemodynamic force*.

### Correlations With Midterm Follow Up

**Figure 2:**
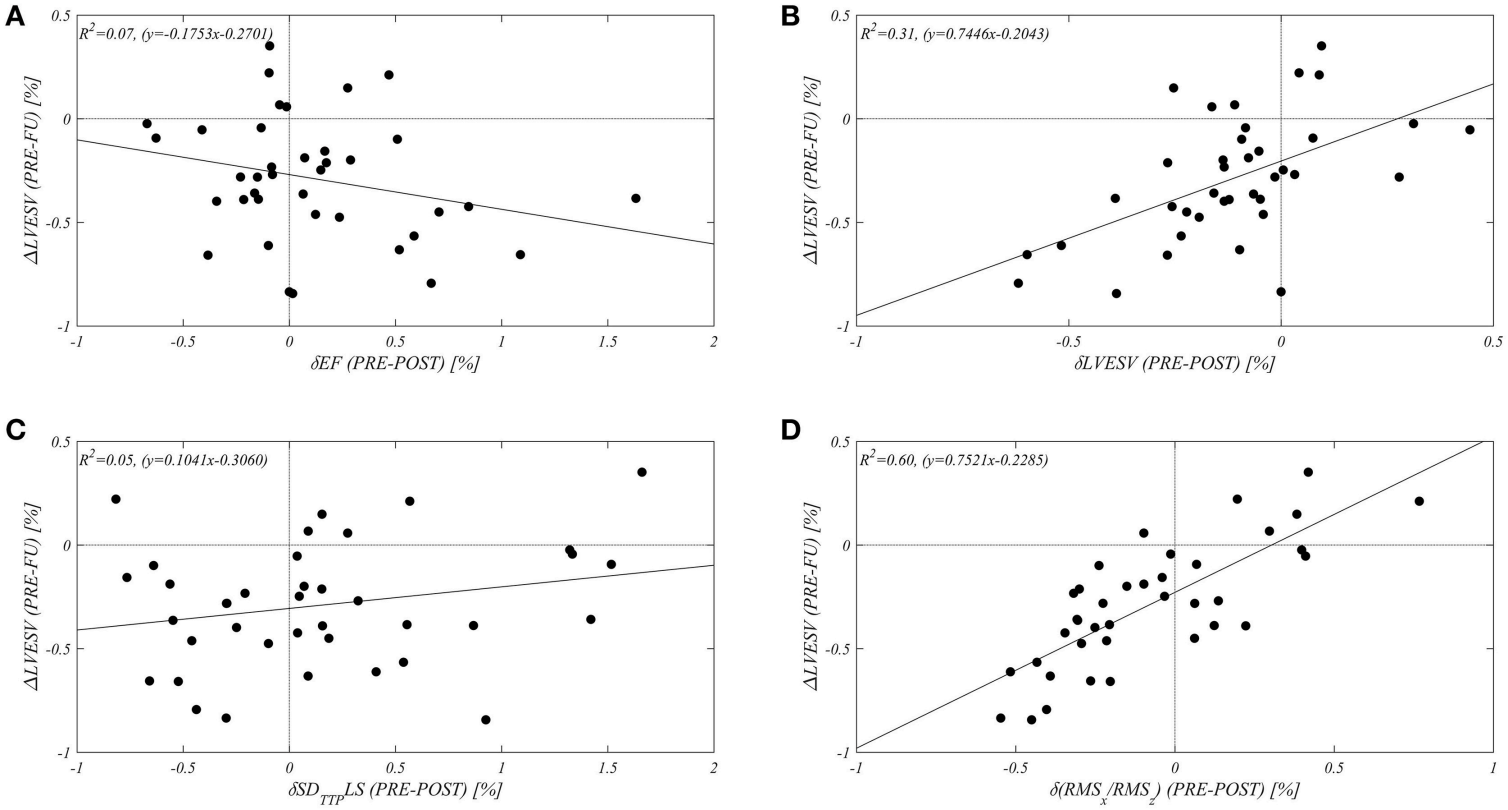
Different Correlations between early changes (expressed by “δ”: from echo PRE CRT implant to echo POST) of selected echocardiographic parameters [**(A)** LVEF, **(B)** LVESV, **(C)** SD_TTP_LS, **(D)** RMSx/RMSz], with relative long term variation of LVESV at midterm follow up(ΔLVESV %).

In order to assess the value of an alignment of HDFs, expressed by RMSx/RMSz, as a potential predictor of responder status at midterm follow up, a correlation study between δ(RMSx/RMSz) and the variation of LVESV at midterm follow up (ΔLVEVS) was performed. This evaluation was compared with respect to the early changes (δ) in LVEF (as a main indicator of LV systolic function), LVESV (to differentiate midterm follow up with respect to early changes), and in SD_TTP_LS (as an indicator of mechanical synchrony). Figure 2 shows the resulting correlation graphs and relative coefficients: in our population δ(RMSx/RMSz) shows the best correlation (*R*^2^ = 0.60) with the variation of LVESV at mid-term follow-up (ΔLVESV). The early change in LVEF and in SD_TTP_LS did not display any correlation with follow up; a small non-significant correlation (*R*^2^ = 0.30) was found between the early (δ) and the midterm follow up (Δ) variation in LVESV (summarized in Table 3).

**Table 3:** Correlations between early changes (“δ”, from PRE to POST implant) of main echocardiographic parameters and difference of LVESV from PRE to follow-up (ΔLVESV).

	***R*^2^**
δLVEF (%)	0,07
δSD_TTP_LS	0,05
δLVESV	0,31
δRMSx/RMSz	0,60

The early decrease in the RMSx/RMSz ratio corresponds to an improved alignment of HDFs after CRT, which represents a measure of the extent of restored physiological LV IVPGs: this correlates with the reduction of LVESV found at a later time. In this perspective, the significance of early change in this ratio was tested with ROC analysis to evaluate if it is predictive of the response status at midterm follow up.

**Figure 3:**
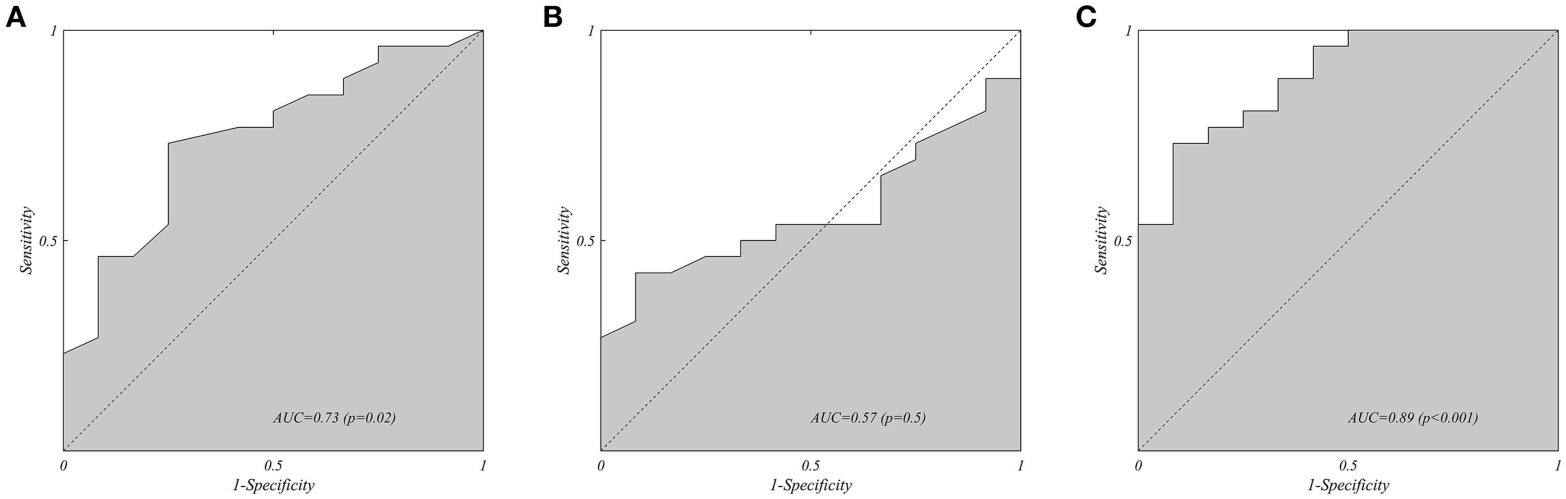
Receiver Operating Characteristic Analysis (ROC) on the all grade Responder Status (R/RS, vs. NR/NegR) at midterm follow, for the hemodynamic force ratio: **(A)** RMSx/RMSz measured PRE implant, **(B)** RMSx/RMSz measured POST implant, and **(C)** early variation δ(RMSx/RMSz) from PRE implant to POST implant. The presence of poor alignment of forces before implant has a mild predictive value for the responder status **(A)**; this does not apply for the value measured after implant **(B)**. Moreover, the improvement of alignment induced by biventricular pacing shows the best predictive value of the eventual responder status at follow up.

As shown in Figure 3C, at ROC analysis the δRMSx/RMSz showed a good accuracy for the prediction of all grade responder status (R/SR) vs. NR/NegR at mid-term follow up (area under the curve (AUC) = 0.891; 95% Confidence Interval (CI) 0.787–0.995, *p* < 0.001). The optimal cut-off of δRMSx/RMSz identified at ROC analysis was −15.1% with a sensitivity and a specificity of 73% and 92%, respectively, for R/SR prediction. Neither the absolute value of this ratio evaluated separately at PRE (Figure 3A, AUC: 0.728, 95% CI 0.557–0.898, *p* = 0,026) nor POST (Figure 3B, AUC 0.569, 95% CI 0.382–0.784, p = 0,5) showed similar predictive accuracy.

### Reproducibility of HDFs Analysis

**Figure 4:**
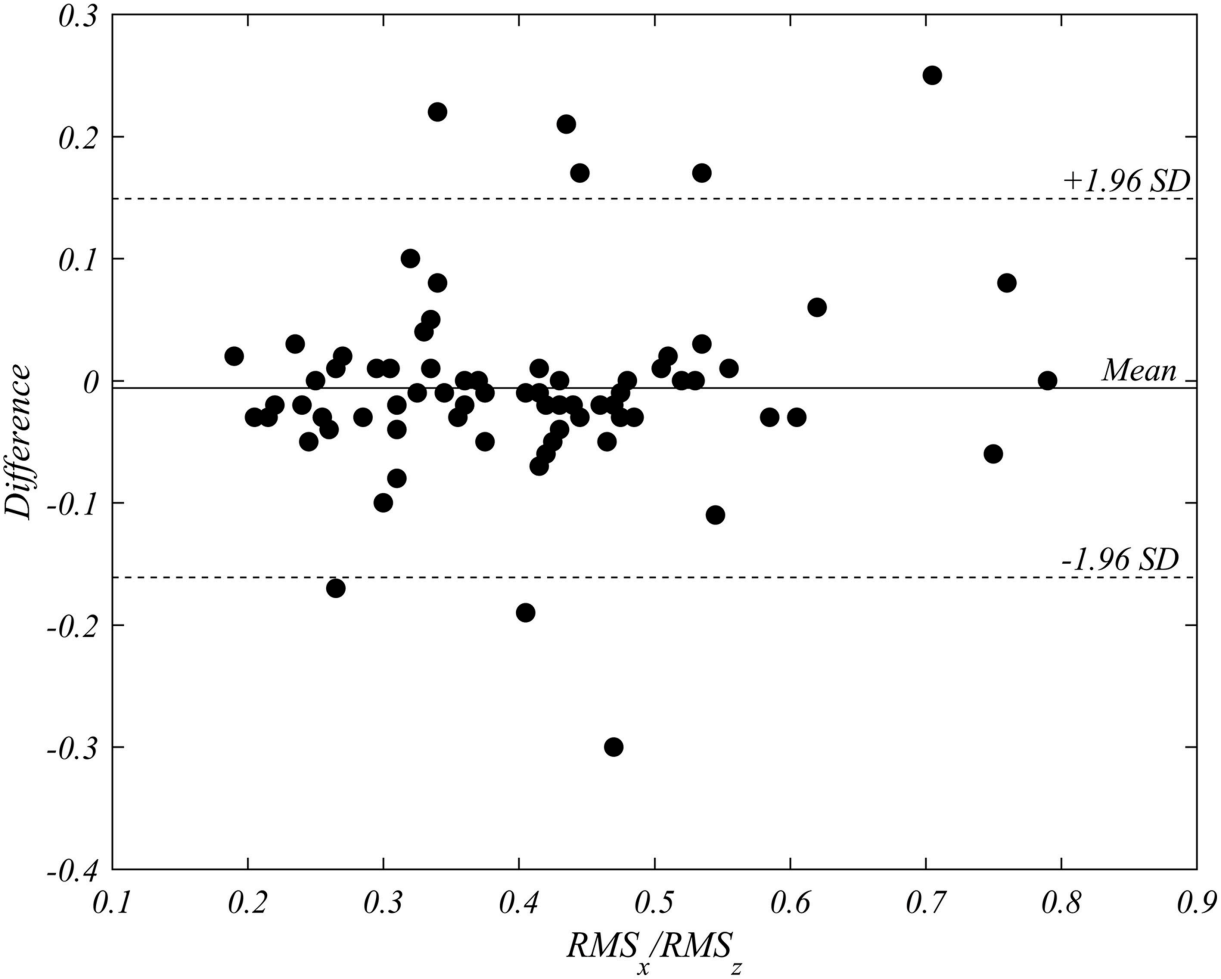
Bland-Altman analysis of inter-operator variability in the triplane measurement of RMSx/RMSz. The analysis performed on all patients in both pre- and post-implant recordings (76 cases), shows the absence of a systematic bias.

The mean of inter-operator difference for the ratio RMSx/RMSz was −0.006 (95% confidence interval −0.02416–0.01206), with *p* = 0.51 in the paired Student *t*-test. Intraclass correlation coefficient (ICC) resulted equal to 0.91 (95% CI: 0.86–0.94). The Bland-Altman analysis, reported in Figure 4, evidences a negligible bias equal to −0.006 and 95% limits of agreement in the interval from −0.161 to 0.149. The reproducibility was statistically satisfactory.

## Discussion

In this pilot, hypotheses-generating retrospective study we demonstrated the feasibility and the potential utility of the sequential echocardiographic assessment of HDFs in CRT recipients as an early marker of resynchronization efficacy at follow-up. To the best of our knowledge, this is the first paper assessing this technique in this setting, and we found it capable to provide us valuable new information about LV function at baseline and early after therapy. In our scenario, this information contributes to a better categorization of patients in terms both of therapy's response and efficacy at follow up.

The main objective of LV function is that of creating flow that, by the law of physics, can develop only in consequence of an intraventricular pressure gradient (IVPG). Normal IVPGs are aligned along the base-apex direction in compliance with the ejection-filling blood motion that occurs from base to apex during systole and vice versa during diastole (8, 9). This natural function means, for example during systole, that the contraction of a region of the LV wall combines with that of facing regions to create a synergistic thrust from the blood pool toward the outflow tract. When this does not occur, as it is in presence of intraventricular dyssynchrony due to LBBB, the contraction of a wall creates a transversal force that overloads the facing regions through the column of fluid (an incompressible medium) between them instead of creating and ejective force. The presence of transversal IVPGs, as it is found in patients with dilated cardiomyopathy (10), thus corresponds to a sub-optimal LV function where the myocardial effort is not functional to ejection and it is spent to stress other regions of the myocardium itself.

Recent 4DFlow studies demonstrated that the increase of the transversal component of HDF with respect to the longitudinal one represents a sensible quantitative marker for the presence of mechanical dyssynchrony (11, 12). Therefore, restoring the natural longitudinal orientation of HDFs corresponds to an improvement of LV synchrony and it is suggested that it may correlate with the favorable response to CRT (14).

This paper applies for the first time a novel method for the estimation of HDFs by regular B-mode images in patients undergoing CRT. This pilot observational study shows, in line with above mentioned preliminary observations (11, 12), that patients with severe LV dysfunction and wide QRS complex show reduced longitudinal HDFs and consequently a relatively increased ratio of transversal/longitudinal HDFs at baseline. A high value of this ratio at baseline witnesses the presence of mechanical dyssynchrony that, in this cohort, is by itself potentially predictive of responder status at midterm follow up (Figure 3A). Moreover, the relative reduction of this ratio induced by pacing is found to have the best predictive accuracy for the efficacy of this treatment at follow up. A growing amount of evidences progressively discovered the importance of the study of myocardial blood flow where vortex formation and HDFs are considered a powerful source of information about LV function (7, 19–21). Despite these suggestions, clinical results are still limited and the applicability of parameters associated to blood dynamics is still uncertain. This is also due to the complexity of techniques for quantifying flow properties in clinical practice. The present results provide an initial evidence for the clinical applicability of a relatively simple method for estimating HDFs parameters (18).

The main net outcome of CRT is ascribed to the responder status at follow up. In the complex world of CRT, many measures of mechanical synchrony have been suggested in order to identify the criteria with highest predictive value (22–24). However, results have been often controversial and non-conclusive (25, 26). One reason could be that synchrony is commonly translated in terms of a requirement of simultaneity for the motion of the LV walls. Simultaneity does not always correspond to an optimal, synergistic dynamics, because the natural LV contraction can present small timing differences that ensure the development of longitudinal IVPGs for the rotatory blood motion due to the vortex. The analysis of HDFs properties may provide more appropriate parameters that correspond to the net result of the many factors concurring to a synchronous LV contraction. In the clinical setting, compared to other techniques of HDFs assessment [e.g., with MRI or echo particle image velocity (Echo-PIV)], the one tested here seems to offer a more easily achievable, reproducible, time and cost saving resource. Furthermore, if confirmed prospectively, the evaluation of HFDs modifications during and early after CRT implantation may be helpful both in eventual optimization of pacing parameters, aimed to obtain a satisfactory reduction of RMSx/RMSz , and in follow up scheduling (a closer visit may be recommended in those patients in which this reduction is not obtained despite pacing optimization). Finally, as previously mentioned, it has to be underlined that the absolute value of this ratio shows *per se* at baseline (PRE) the potential to predict a higher probability of favorable response to CRT: in respect to the current claim for more accurate selection criteria in CRT recipients (5), this aspect deserve further evaluation in future prospective studies.

### Study Limitations

The present study was performed retrospectively on a small sample of patients. Thus, our preliminary results should be considered as proof of concept and hypotheses generating, future larger prospectives investigations are required to explore more extensively the applicability and the advantages of this methodology in candidates to CRT. The time intervals between PRE and POST echocardiographic evaluations were slightly different among patients. However, in our opinion the limit of 2 months avoided that the progression of the underlying disease, rather than the net effect of biventricular pacing, might have influenced HDFs. With respect to the optimization of the recording protocol, the echocardiographic exams PRE and POST implant were performed without considering the need of a comparative assessment, thus a variability may also be partly imputable to small changes in the acquisition practice including different operators.

This study was aimed to provide an initial evaluation of an approach based on HDFs for the prediction of response to CRT. This preliminary work was required for verifying whether this approach could deserve the design of a prospective clinical study in the same subject.

## Conclusions

This pilot observational study demonstrated that the realignment of LV HDFs induced by biventricular pacing, assessed through a new echocardiographic technique early after implantation, correlates with LV reverse remodeling at follow-up in HFrEF patients treated with CRT. These results should encourage the design of a larger prospective clinical study to verify the potential of systematic HDFs assessment in selecting the optimal candidates to CRT and in guiding pacing optimization.

## Data Availability

The raw data supporting the conclusions of this manuscript will be made available by the authors, without undue reservation, to any qualified researcher.

## Ethics Statement

This study was carried out in accordance with the recommendations of our clinical institution, the Azienda Ospedaliera Universitaria Integrata of Trieste. All subjects gave written informed consent in accordance with the Declaration of Helsinki. The protocol was approved by the Institutional Review Board of the Azienda Ospedaliera Universitaria Integrata of Trieste.

## Author Contributions

All authors listed have made a substantial, direct and intellectual contribution to the work, and approved it for publication.

### Conflict of Interest Statement

GP is a shareholder of AMID SRL, which is a partner of TomTec Gmbh. The remaining authors declare that the research was conducted in the absence of any commercial or financial relationships that could be construed as a potential conflict of interest.
